# Correction to: Consolidated Health Economic Evaluation Reporting Standards 2022 (CHEERS 2022) Statement: Updated Reporting Guidance for Health Economic Evaluations

**DOI:** 10.1007/s40258-022-00743-y

**Published:** 2022-07-15

**Authors:** Don Husereau, Michael Drummond, Federico Augustovski, Esther de Bekker-Grob, Andrew H. Briggs, Chris Carswell, Lisa Caulley, Nathorn Chaiyakunapruk, Dan Greenberg, Elizabeth Loder, Josephine Mauskopf, C. Daniel Mullins, Stavros Petrou, Raoh-Fang Pwu, Sophie Staniszewska

**Affiliations:** 1 School of Epidemiology and Public Health, University of Ottawa, Ottawa, ON Canada; 2 Institute of Health Economics, Edmonton, AL Canada; 3 Centre for Health Economics, University of York, York, UK; 4 Health Technology Assessment and Health Economics Department of the Institute for Clinical Effectiveness and Health Policy (IECS-CONICET), Buenos Aires, Argentina; 5 University of Buenos Aires, Buenos Aires, Argentina; 6 CONICET (National Scientific and Technical Research Council), Buenos Aires, Argentina; 7 Erasmus School of Health Policy and Management, Erasmus University Rotterdam, Rotterdam, The Netherlands; 8 London School of Hygiene and Tropical Medicine, London, England; 9 Adis Journals, Springer Nature, Auckland, New Zealand; 10 Department of Otolaryngology-Head and Neck Surgery, University of Ottawa, Ottawa, ON Canada; 11 Clinical Epidemiology Program and Center for Journalology, Ottawa Hospital Research Institute, Ottawa, ON Canada; 12 Department of Epidemiology, Erasmus University Medical Center Rotterdam, Rotterdam, The Netherlands; 13 Department of Pharmacotherapy, College of Pharmacy, University of Utah, Salt Lake City, UT USA; 14 Department of Health Policy and Management, School of Public Health, Faculty of Health Sciences, Ben-Gurion University of the Negev, Be’er-Sheva, Israel; 15 Harvard Medical School, Boston, MA USA; 16 The BMJ, London, UK; 17 RTI Health Solutions, RTI International, Research Triangle Park, NC USA; 18 School of Pharmacy, University of Maryland Baltimore, Baltimore, MD USA; 19 Nuffield Department of Primary Care Health Sciences, University of Oxford, Oxford, UK; 20 National Hepatitis C Program Office, Ministry of Health and Welfare, Taipei City, Taiwan; 21 Warwick Research in Nursing, University of Warwick Warwick Medical School, Warwick, UK

## Correction to: Applied Health Economics and Health Policy (2022) 20:213–221 10.1007/s40258-021-00704-x

This article was published under the incorrect license (CC-BY-NC). The correct license is CC-BY.

This article is licensed under a Creative Commons Attribution 4.0 International License, which permits use, sharing, adaptation, distribution and reproduction in any medium or format, as long as you give appropriate credit to the original author(s) and the source, provide a link to the Creative Commons licence, and indicate if changes were made. The images or other third-party material in this article are included in the article's Creative Commons licence, unless indicated otherwise in a credit line to the material. If material is not included in the article's Creative Commons licence and your intended use is not permitted by statutory regulation or exceeds the permitted use, you will need to obtain permission directly from the copyright holder. To view a copy of this licence, visit http://creativecommons.org/licenses/by/4.0/.

The original article has been corrected.

